# The genome sequence of the meadow brown, *Maniola jurtina *(Linnaeus, 1758)

**DOI:** 10.12688/wellcomeopenres.17304.1

**Published:** 2021-11-05

**Authors:** Konrad Lohse, Jamie Weir, Darwin Tree of Life Barcoding collective, Wellcome Sanger Institute Tree of Life programme, Wellcome Sanger Institute Scientific Operations: DNA Pipelines collective, Tree of Life Core Informatics collective, Darwin Tree of Life Consortium

**Affiliations:** 1 Institute of Evolutionary Biology, University of Edinburgh, Edinburgh, UK

**Keywords:** Maniola jurtina, meadow brown, genome sequence, chromosomal

## Abstract

We present a genome assembly from an individual female *Maniola jurtina *(the meadow brown; Arthropoda; Insecta; Lepidoptera; Nymphalidae). The genome sequence is 402 megabases in span. The complete assembly is scaffolded into 30 chromosomal pseudomolecules, with the W and Z sex chromosome assembled. Gene annotation of this assembly on Ensembl has identified 12,502 protein coding genes.

## Species taxonomy

Eukaryota; Metazoa; Ecdysozoa; Arthropoda; Hexapoda; Insecta; Pterygota; Neoptera; Endopterygota; Lepidoptera; Glossata; Ditrysia; Papilionoidea; Nymphalidae; Satyrinae; Satyrini; Maniolina; Maniola; *Maniola jurtina* (Linnaeus, 1758) (NCBI:txid191418).

## Introduction

The meadow brown *Maniola jurtina* is a common, Palearctic butterfly occurring throughout Europe, the Middle East, and North Africa ( gbif.org, 2021). Both widespread and often abundant, the species is associated with almost any grassy habitats ( South, 1906), reaching highest densities in areas where grazing or other pressures keep the sward at an intermediate height ( Maitland Emmet & Heath, 1989). Although *M. jurtina* is consistently univoltine, emergence occurs over a prolonged period in summer, which varies in length geographically and with habitat types ( Brakefield, 1987). In many Mediterranean populations, females aestivate during the hottest months of the year ( Scali, 1971). Eggs are laid singly or in small clusters, both on individual blades of grass or loose into a suitable tuft ( Maitland Emmet & Heath, 1989), preferentially on *Poa*, *Agrostis* and *Lolium*. Larvae overwinter, but do not undergo true diapause, and feed intermittently in warm spells. Pupae show considerable variation in colouration which is affected by light and temperature ( Brakefield, 1979). The species exhibits a great deal of phenotypic variation both within and between populations ( Thomson, 1969). Four sub-species are known from the British Isles – ssp. *splendida, insularis*, *iernes*, and *cassiteridum* – although the validity of these taxa is questionable ( Weir & Others, 2016), since they seem to be phenotypic extremes at opposing ends of clines ( Maitland Emmet & Heath, 1989). In their pioneering work in ecological genetics, Ford and Dowdeswell considered the evolutionary factors shaping variation in the spot patterning of the underside of the hindwings in *M. jurtina*, initially on the Isles of Scilly, then the British mainland (reviewed in ( Ford, 1964) and ( Dowdeswell, 1981). Several early studies ( Bigger, 1960; Federley, 1938; Lorković, 1941), summarised in ( Robinson, 1971), report a karyotype of 29 chromosomes. The genome size has been estimated as 367.3 Mb ( Mackintosh *et al.*, 2019). We note the publication of a *de novo* genome assembly of *M. jurtina* by ( Singh *et al.*, 2020) and believe that the sequence described here, generated as part of the Darwin Tree of Life project, will further aid understanding of the biology of this butterfly.

## Genome sequence report

**Figure 1.**
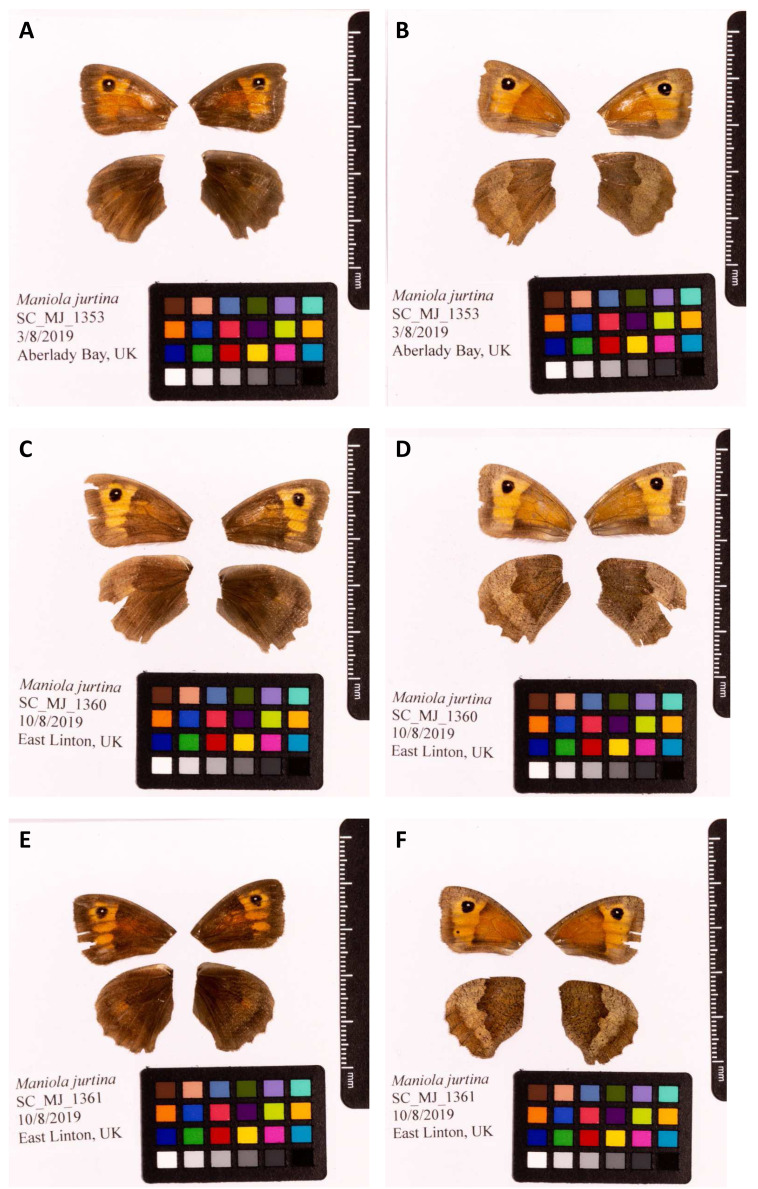
Fore and hind wings of *Maniola jurtina* specimens from which the genome was sequenced. ( **A**) Dorsal surface view of wings from specimen SC_MJ_1353 (ilManJurt1) from Aberlady, Scotland, UK, used to generate Pacific Biosciences and 10X data. ( **B**) Ventral surface view of wings from specimen SC_MJ_1353 (ilManJurt1) from Aberlady, Scotland, UK, used to generate Pacific Biosciences and 10X data. ( **C**) Dorsal surface view of wings from specimen SC_MJ_1360 (ilManJurt2) from East Linton, Scotland, UK, used to generate RNA-Seq data. ( **D**) Ventral surface view of wings from specimen SC_MJ_1360 (ilManJurt2) from East Linton, Scotland, UK, used to generate RNA-Seq data. ( **E**) Dorsal surface view of wings from specimen SC_MJ_1361 (ilManJurt3) from East Linton, Scotland, UK, used to generate Hi-C data. ( **F**) Ventral surface view of wings from specimen SC_MJ_1361 (ilManJurt3) from East Linton, Scotland, UK, used to generate Hi-C data.

The genome was sequenced from a female *M. jurtina* (ilManJurt1; Figure 1A, B) collected from Aberlady Bay, East Lothian, Scotland, UK (latitude 56.019964, longitude -2.85808). Hi-C data were generated from another individual (ilManJurt3; Figure 1E, F) collected from East Linton, East Lothian, Scotland, UK (latitude 55.977161, longitude -2.667545). A total of 76-fold coverage in Pacific Biosciences single-molecule long reads (N50 14 kb) and 88-fold coverage in 10X Genomics read clouds were generated. Primary assembly contigs were scaffolded with chromosome conformation Hi-C data. Manual assembly curation corrected 24 missing/misjoins and removed two haplotypic duplications, reducing the assembly size by 1.67% and scaffold number by 24.39%.

**Figure 2.**
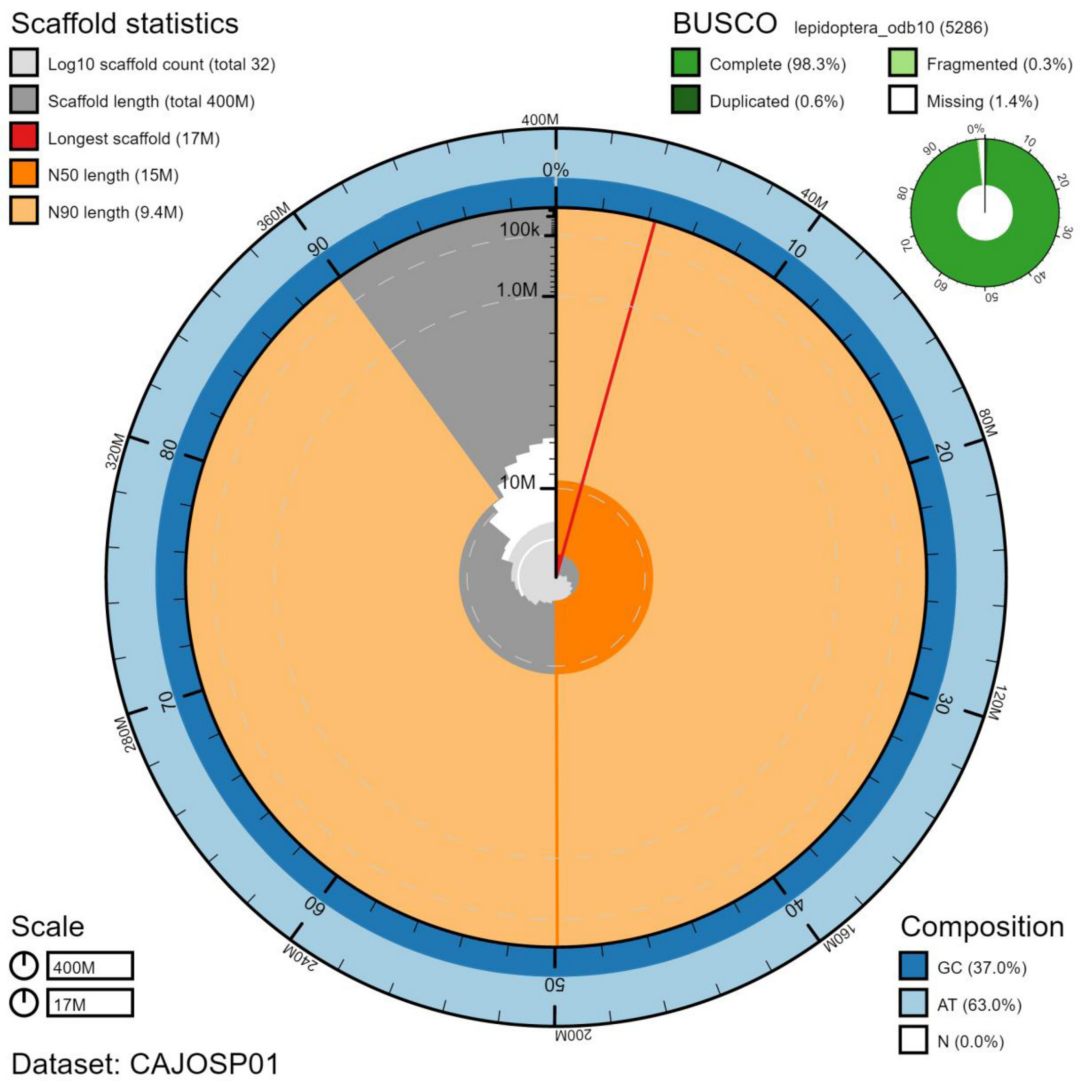
Genome assembly of *Maniola jurtina*, ilManJurt1.1: metrics. The BlobToolKit Snailplot shows N50 metrics and BUSCO gene completeness. The main plot is divided into 1,000 size-ordered bins around the circumference with each bin representing 0.1% of the 402,054,102 bp assembly. The distribution of chromosome lengths is shown in dark grey with the plot radius scaled to the longest chromosome present in the assembly (17,318,487 bp, shown in red). Orange and pale-orange arcs show the N50 and N90 chromosome lengths (15,090,120 and 9,430,113 bp), respectively. The pale grey spiral shows the cumulative chromosome count on a log scale with white scale lines showing successive orders of magnitude. The blue and pale-blue area around the outside of the plot shows the distribution of GC, AT and N percentages in the same bins as the inner plot. A summary of complete, fragmented, duplicated and missing BUSCO genes in the lepidoptera_odb10 set is shown in the top right. An interactive version of this figure is available at https://blobtoolkit.genomehubs.org/view/ilManJurt1.1/dataset/CAJOSP01/snail.

The final assembly has a total length of 402 Mb in 30 sequence scaffolds with a scaffold N50 of 15 Mb ( Table 1). Of the assembly sequence, 100% was assigned to 30 chromosomal-level scaffolds, representing 28 autosomes (numbered by sequence length), and the W and Z sex chromosome ( Figure 2– Figure 5; Table 2). The assembly has a BUSCO ( Simão *et al.*, 2015) completeness of 98.3% using the lepidoptera_odb9 reference set. While not fully phased, the assembly deposited is of one haplotype. Contigs corresponding to the second haplotype have also been deposited.

**Table 1.** Genome data for *Maniola jurtina*, ilManJurt1.1.

*Project accession data*	
Assembly identifier	ilManJurt1.1
Species	*Maniola jurtina*
Specimen	ilManJurt1 (genome assembly); ilManJurt2, ilManJurt5 (RNA-Seq); ilManJurt3 (Hi-C)
NCBI taxonomy ID	NCBI:txid191418
BioProject	PRJEB43535
BioSample ID	SAMEA7523158
Isolate information	Female, whole organisms
*Raw data accessions*	
PacificBiosciences SEQUEL II	ERR6576323
10X Genomics Illumina	ERR6054518–ERR6054521
Hi-C Illumina	ERR6054522
Illumina PolyA RNAseq	ERR6054523, ERR6787422
*Genome assembly*	
Assembly accession	GCA_905333055.1
*Accession of alternate haplotype*	GCA_905333105.1
Span (Mb)	402
Number of contigs	53
Contig N50 length (Mb)	13
Number of scaffolds	32
Scaffold N50 length (Mb)	15
Longest scaffold (Mb)	17
BUSCO * genome score	C:98.3%[S:97.7%,D:0.6%],F:0.3%,M:1.4%,n:5286

*BUSCO scores based on the lepidoptera_odb10 BUSCO set using v5.1.2. C= complete [S= single copy, D=duplicated], F=fragmented, M=missing, n=number of orthologues in comparison. A full set of BUSCO scores is available at https://blobtoolkit.genomehubs.org/view/ilManJurt1.1/dataset/CAJOSP01/busco.

**Figure 3.**
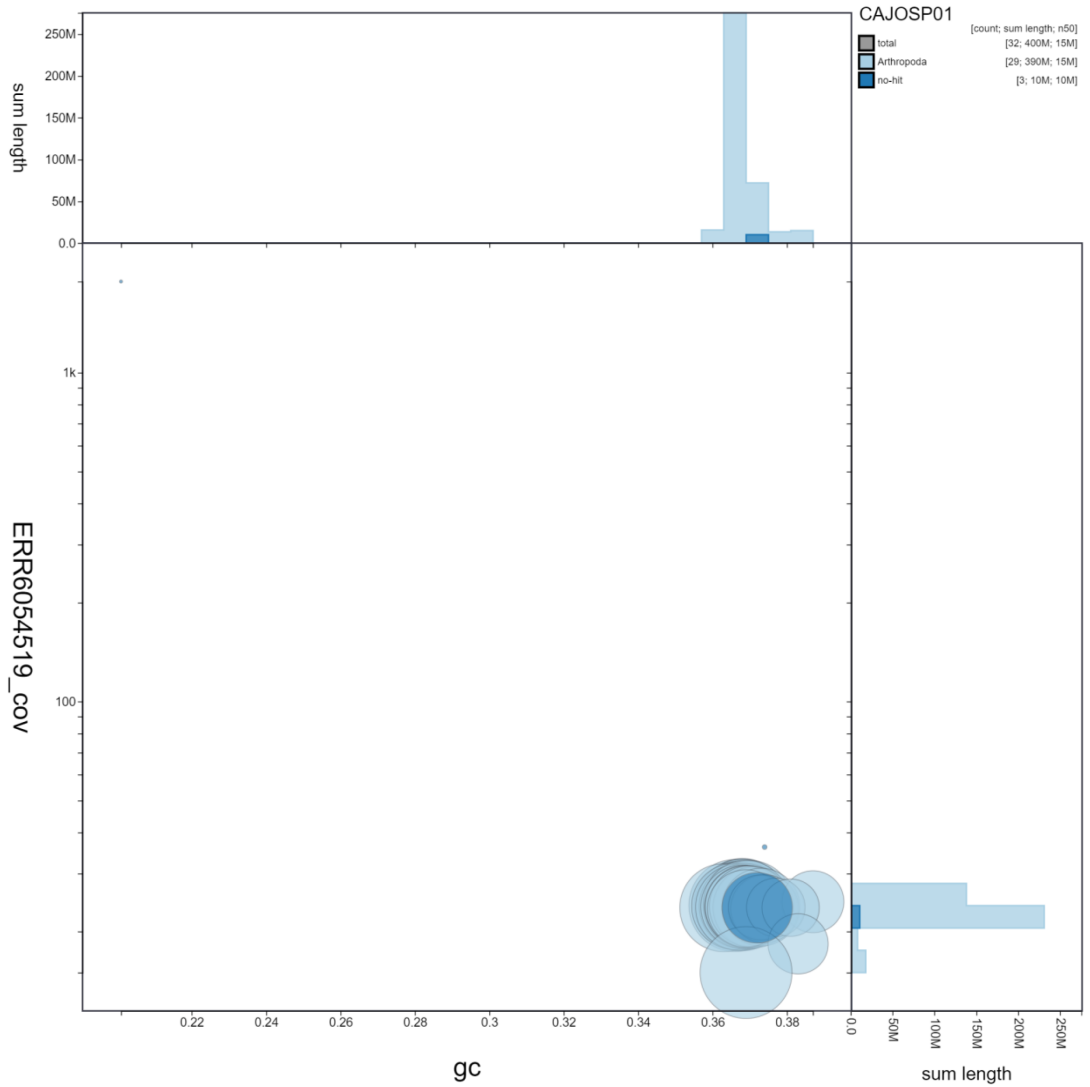
Genome assembly of *Maniola jurtina*, ilManJurt1.1: GC coverage. BlobToolKit GC-coverage plot. Chromosomes are coloured by phylum. Circles are sized in proportion to chromosome length. Histograms show the distribution of scaffold length sum along each axis. An interactive version of this figure is available at https://blobtoolkit.genomehubs.org/view/ilManJurt1.1/dataset/CAJOSP01/blob.

**Figure 4.**
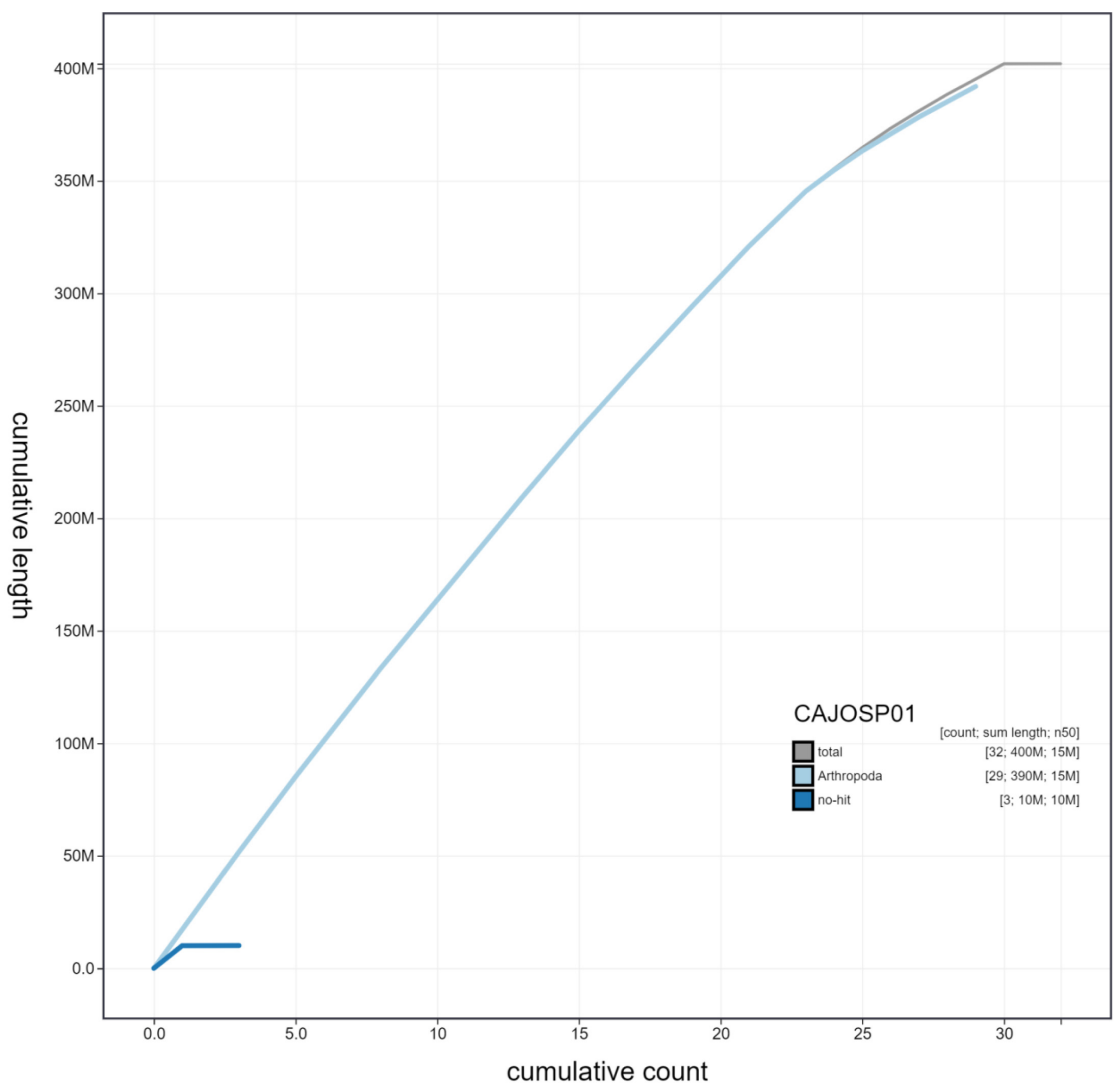
Genome assembly of *Maniola jurtina*, ilManJurt1.1: cumulative sequence. BlobToolKit cumulative sequence plot. The grey line shows cumulative length for all chromosomes. Coloured lines show cumulative lengths of chromosomes assigned to each phylum using the buscogenes taxrule. An interactive version of this figure is available at https://blobtoolkit.genomehubs.org/view/ilManJurt1.1/dataset/CAJOSP01/cumulative.

**Figure 5.**
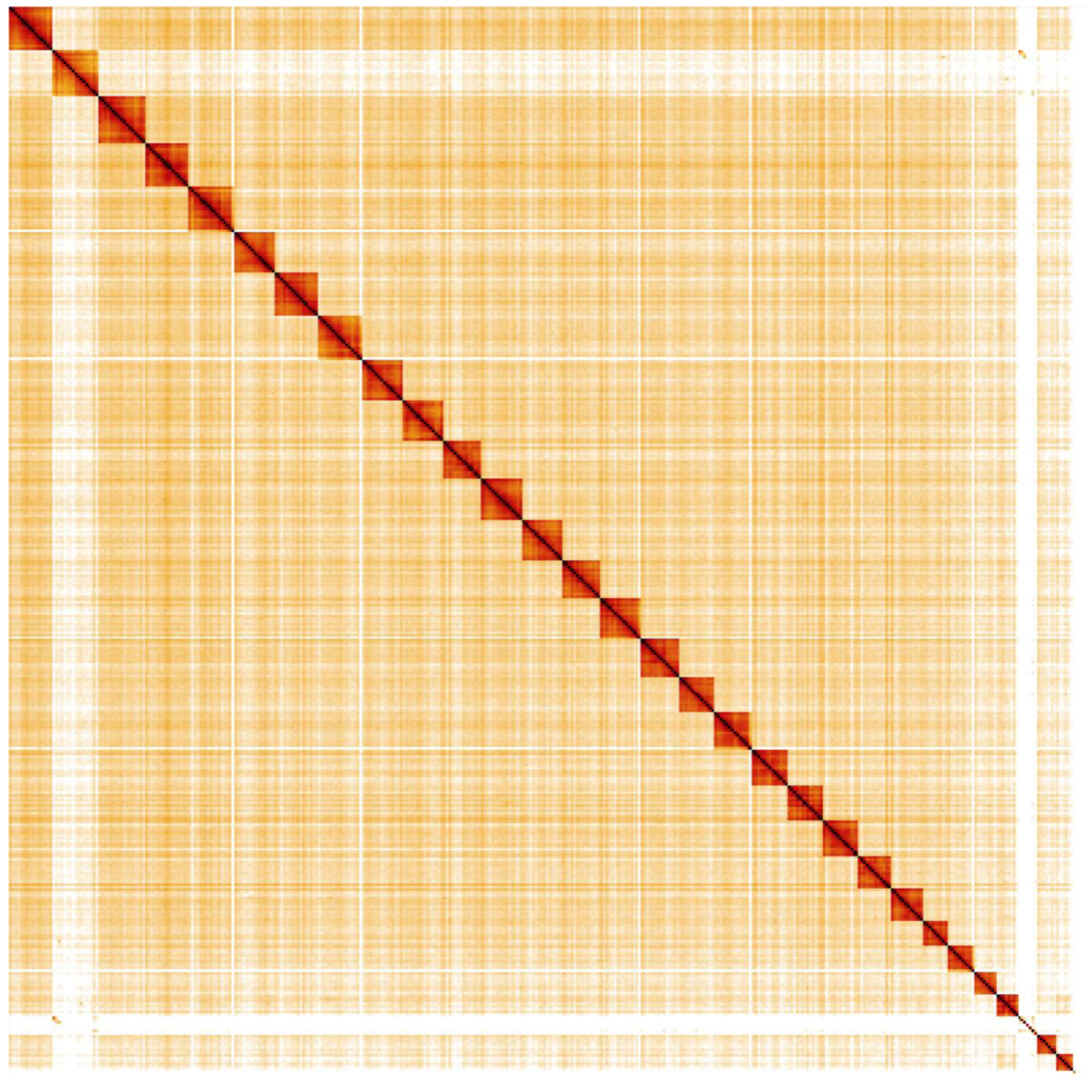
Genome assembly of *Maniola jurtina*, ilManJurt1.1: Hi-C contact map. Hi-C contact map of the ilManJurt1.1 assembly, visualised in HiGlass. Chromosomes are shown in size order from left to right and top to bottom.

## Gene annotation

The Ensembl gene annotation system ( Aken *et al.*, 2016) was used to generate annotation for the *Maniola jurtina* assembly (GCA_905333055.1, see https://rapid.ensembl.org/Maniola_jurtina_GCA_905333055.1/; Table 1). The annotation was created primarily through alignment of transcriptomic data to the genome, with gap filling via protein to genome alignments of a select set of proteins from UniProt ( UniProt Consortium, 2019) and OrthoDB ( Kriventseva *et al.*, 2008). Prediction tools, CPC2 ( Kang *et al.*, 2017) and RNAsamba ( Camargo *et al.*, 2020), were used to aid determination of protein coding genes.

**Table 2.** Chromosomal pseudomolecules in the genome assembly of *Maniola jurtina*, ilManJurt1.1.

INSDC accession	Chromosome	Size (Mb)	GC%
HG995207.1	1	17.32	36.8
HG995209.1	2	17.19	36.6
HG995210.1	3	16.76	36.8
HG995211.1	4	16.67	36.8
HG995212.1	5	16.23	36.7
HG995213.1	6	16.20	36.8
HG995214.1	7	15.71	36.3
HG995215.1	8	15.42	36.9
HG995216.1	9	15.23	37
HG995217.1	10	15.17	36.6
HG995218.1	11	15.11	36.8
HG995219.1	12	15.09	36.7
HG995220.1	13	14.83	36.9
HG995221.1	14	14.70	36.9
HG995222.1	15	14.15	36.9
HG995223.1	16	14.03	36.8
HG995224.1	17	13.72	36.9
HG995225.1	18	13.54	36.9
HG995226.1	19	13.29	37.1
HG995227.1	20	13.28	37
HG995228.1	21	12.26	37.3
HG995229.1	22	12.13	36.9
HG995230.1	23	10.05	37.2
HG995231.1	24	9.43	37.4
HG995232.1	25	8.57	37.3
HG995233.1	26	7.75	38.7
HG995235.1	27	6.95	37.7
HG995236.1	28	6.66	38.1
HG995234.1	W	7.35	38.3
HG995208.1	Z	17.21	36.9
HG995237.1	MT	0.02	20.3
-	Unplaced	0.04	37.4

## Methods

### Sample acquisition and nucleic acid extraction

Four female *M. jurtina* samples (genome assembly, ilManJurt1; RNAseq, ilManJurt2, ilManJurt5; Hi-C, ilManJurt3) were collected and used for sequencing. Sample ilManJurt1 was caught in Aberlady, East Lothian, UK (latitude 56.019964, longitude -2.85808). Samples ilManJurt2 ( Figure 1C, D), ilManJurt3 and ilManJurt5 were caught in East Linton, East Lothian, UK (latitude 56.019964, longitude -2.85808). All samples were collected using a handnet by Konrad Lohse, University of Edinburgh, and snap-frozen in liquid nitrogen.

DNA was extracted at the Wellcome Sanger Institute (WSI) Scientific Operations core from the whole organism using the Qiagen MagAttract HMW DNA kit, according to the manufacturer’s instructions. RNA was extracted in the Tree of Life Laboratory at the WSI using TRIzol (Invitrogen), according to the manufacturer’s instructions. RNA was then eluted in 50 μl RNAse-free water and its concentration assessed using a Nanodrop spectrophotometer and Qubit Fluorometer using the Qubit RNA Broad-Range (BR) Assay kit. Analysis of the integrity of the RNA was done using Agilent RNA 6000 Pico Kit and Eukaryotic Total RNA assay.

### Sequencing

Pacific Biosciences HiFi circular consensus and 10X Genomics Chromium read cloud sequencing libraries were constructed according to the manufacturers’ instructions. Poly(A) RNA-Seq libraries were constructed using the NEB Ultra II RNA Library Prep kit. Sequencing was performed by the Scientific Operations core at the Wellcome Sanger Institute on Pacific Biosciences SEQUEL II (HiFi), Illumina HiSeq X (10X) and Illumina HiSeq 4000 (RNA-Seq) instruments. Hi-C data were generated using the Qiagen EpiTect Hi-C kit and sequenced on HiSeq X.

### Genome assembly

Assembly was carried out with Hifiasm ( Cheng *et al.*, 2021). Haplotypic duplication was identified and removed with purge_dups ( Guan *et al.*, 2020). One round of polishing was performed by aligning 10X Genomics read data to the assembly with longranger align, calling variants with freebayes ( Garrison & Marth, 2012). The assembly was then scaffolded with Hi-C data ( Rao *et al.*, 2014) using SALSA2 ( Ghurye *et al.*, 2019). The assembly was checked for contamination and corrected using the gEVAL system ( Chow *et al.*, 2016) as described previously ( Howe *et al.*, 2021). Manual curation ( Howe *et al.*, 2021) was performed using gEVAL, HiGlass ( Kerpedjiev *et al.*, 2018) and Pretext. The mitochondrial genome was assembled using MitoHiFi ( Uliano-Silva *et al.*, 2021). The genome was analysed and BUSCO scores generated within the BlobToolKit environment ( Challis *et al.*, 2020). Table 3 contains a list of all software tool versions used, where appropriate.

**Table 3.** Software tools used.

Software tool	Version	Source
Hifiasm	0.12	Cheng *et al.*, 2021
purge_dups	1.2.3	Guan *et al.*, 2020
SALSA2	2.2	Ghurye *et al.*, 2019
longranger align	2.2.2	https://support.10xgenomics.com/genome-exome/software/pipelines/latest/advanced/other-pipelines
freebayes	v1.3.1-17-gaa2ace8	Garrison & Marth, 2012
MitoHiFi	1.0	https://github.com/marcelauliano/MitoHiFi
gEVAL	N/A	Chow *et al.*, 2016
HiGlass	1.11.6	Kerpedjiev *et al.*, 2018
PretextView	0.1.x	https://github.com/wtsi-hpag/PretextView
BlobToolKit	2.6.2	Challis *et al.*, 2020

### Ethical/compliance issues

The materials that have contributed to this genome note were supplied by a Tree of Life collaborator. The Wellcome Sanger Institute employs a process whereby due diligence is carried out proportionate to the nature of the materials themselves, and the circumstances under which they have been/are to be collected and provided for use. The purpose of this is to address and mitigate any potential legal and/or ethical implications of receipt and use of the materials as part of the research project, and to ensure that in doing so we align with best practice wherever possible.

The overarching areas of consideration are:

- Ethical review of provenance and sourcing of the material;
- Legality of collection, transfer and use (national and international).

Each transfer of samples is undertaken according to a Research Collaboration Agreement or Material Transfer Agreement entered into by the Tree of Life collaborator, Genome Research Limited (operating as the Wellcome Sanger Institute) and in some circumstances other Tree of Life collaborators.

## Data availability

European Nucleotide Archive: Maniola jurtina (meadow brown). Accession number PRJEB43535; https://identifiers.org/ena.embl/PRJEB43535.

The genome sequence is released openly for reuse. The *M. jurtina* genome sequencing initiative is part of the Darwin Tree of Life (DToL) project. All raw sequence data and the assembly have been deposited in INSDC databases. Raw data and assembly accession identifiers are reported in Table 1.

## Author information

Members of the Darwin Tree of Life Barcoding collective are listed here: https://doi.org/10.5281/zenodo.4893704.

Members of the Wellcome Sanger Institute Tree of Life programme collective are listed here: https://doi.org/10.5281/zenodo.5377053.

Members of Wellcome Sanger Institute Scientific Operations: DNA Pipelines collective are listed here: https://doi.org/10.5281/zenodo.4790456.

Members of the Tree of Life Core Informatics collective are listed here: https://doi.org/10.5281/zenodo.5013542.

Members of the Darwin Tree of Life Consortium are listed here: https://doi.org/10.5281/zenodo.4783559.

## References

[ref-1] Aken BL Ayling S Barrell D : The Ensembl Gene Annotation System. *Database (Oxford).* 2016;baw093. 10.1093/database/baw093 27337980 PMC4919035

[ref-2] Bigger TRL : Chromosome Numbers of Lepidoptera. Part I. *Entomologist’s Gazette.* 1960;11:149–52.

[ref-3] Brakefield PM : An Experimental Study of the Maintenance of Variation in Spot Pattern in Maniola Jurtina. Thesis Ph.D., Liverpool University.1979.

[ref-4] Brakefield PM : Geographical Variability In, and Temperature Effects On, the Phenology of Maniola Jurtina and Pyronia Tithonus (Lepidoptera, Satyrinae) in England and Wales. *Ecol Entomol.* 1987;12(2):139–48. Reference Source

[ref-5] Camargo AP Sourkov V Pereira GAG : RNAsamba: Neural Network-Based Assessment of the Protein-Coding Potential of RNA Sequences. *NAR Genom Bioinform.* 2020;2(1):lqz024. 10.1093/nargab/lqz024 33575571 PMC7671399

[ref-6] Challis R Richards E Rajan J : BlobToolKit - Interactive Quality Assessment of Genome Assemblies. *G3 (Bethesda).* 2020;10(4):1361–74. 10.1534/g3.119.400908 32071071 PMC7144090

[ref-7] Cheng H Concepcion GT Feng X : Haplotype-Resolved de Novo Assembly Using Phased Assembly Graphs with Hifiasm. *Nat Methods.* 2021;18(2):170–75. 10.1038/s41592-020-01056-5 33526886 PMC7961889

[ref-8] Chow W Brugger K Caccamo M : gEVAL - a web-based browser for evaluating genome assemblies. *Bioinformatics.* 2016;32(16):2508–10. 10.1093/bioinformatics/btw159 27153597 PMC4978925

[ref-9] Dowdeswell WH : The Life of the Meadow Brown. Heinemann Educational Books.1981. Reference Source

[ref-10] Federley H : Chromosomenzahlen Finnlän-Discher Lepidopteren. *Hereditas.* 1938;24(4):397–464. 10.1111/j.1601-5223.1938.tb03219.x

[ref-11] Ford EB : Ecological Genetics. Metheun.1964. Reference Source

[ref-12] Garrison E Marth G : Haplotype-Based Variant Detection from Short-Read Sequencing. arXiv: 1207.3907.2012. Reference Source

[ref-13] gbif.org, Registry-Migration: GBIF Backbone Taxonomy.GBIF Secretariat.2021. 10.15468/39OMEI

[ref-14] Ghurye J Rhie A Walenz BP : Integrating Hi-C Links with Assembly Graphs for Chromosome-Scale Assembly. *PLoS Comput Biol.* 2019;15(8):e1007273. 10.1371/journal.pcbi.1007273 31433799 PMC6719893

[ref-15] Guan D McCarthy SA Wood J : Identifying and removing haplotypic duplication in primary genome assemblies. *Bioinformatics.* 2020;36(9):2896–2898. 10.1093/bioinformatics/btaa025 31971576 PMC7203741

[ref-16] Howe K Chow W Collins J : Significantly Improving the Quality of Genome Assemblies through Curation. *GigaScience.* 2021;10(1):giaa153. 10.1093/gigascience/giaa153 33420778 PMC7794651

[ref-17] Kang YJ Yang DC Kong L : CPC2: A Fast and Accurate Coding Potential Calculator Based on Sequence Intrinsic Features. *Nucleic Acids Res.* 2017;45(W1):W12–16. 10.1093/nar/gkx428 28521017 PMC5793834

[ref-18] Kerpedjiev P Abdennur N Lekschas F : HiGlass: Web-Based Visual Exploration and Analysis of Genome Interaction Maps. *Genome Biol.* 2018;19(1):125. 10.1186/s13059-018-1486-1 30143029 PMC6109259

[ref-19] Kriventseva EV Rahman N Espinosa O : OrthoDB: The Hierarchical Catalog of Eukaryotic Orthologs. *Nucleic Acids Res.* 2008;36(Database issue):D271–75. 10.1093/nar/gkm845 17947323 PMC2238902

[ref-20] Lorković Z : Die Chromosomenzahlen in der Spermatogenese der Tagfalter. *Chromosoma.* 1941;2(1):155–91. 10.1007/BF00325958

[ref-21] Mackintosh A Laetsch DR Hayward A : The Determinants of Genetic Diversity in Butterflies. *Nat Commun.* 2019;10(1):3466. 10.1038/s41467-019-11308-4 31371715 PMC6672018

[ref-22] Maitland Emmet AM Heath J : The Moths and Butterflies of Great Britain and Ireland: Hesperiidae - Nymphalidae, the Butterflies.Harley Books,1989. Reference Source

[ref-23] Rao SSP Huntley MH Durand NC : A 3D Map of the Human Genome at Kilobase Resolution Reveals Principles of Chromatin Looping. *Cell.* 2014;159(7):1665–80. 10.1016/j.cell.2014.11.021 25497547 PMC5635824

[ref-24] Robinson R : Lepidoptera Genetics.Pergamon Press,1971. 10.1016/C2013-0-01588-5

[ref-25] Scali V : SPOT DISTRIBUTION IN MANIOLA JURTINA (L.) (LEPIDOPTERA SATYRIDAE): TUSCAN MAINLAND 1967--1969. *Monitore Zoologico Italiano-Italian Journal of Zoology.* 1971;5(3):147–63. Reference Source

[ref-26] Simão FA Waterhouse RM Ioannidis P : BUSCO: Assessing Genome Assembly and Annotation Completeness with Single-Copy Orthologs. *Bioinformatics.* 2015;31(19):3210–12. 10.1093/bioinformatics/btv351 26059717

[ref-27] Singh KS Hosken DJ Wedell N : *De Novo* Genome Assembly of the Meadow Brown Butterfly, *Maniola Jurtina.* *G3 (Bethesda).* 2020;10(5):1477–84. 10.1534/g3.120.401071 32161089 PMC7202024

[ref-28] South R : The Butterflies of the British Isles.Frederick Warne & Co,1906. Reference Source

[ref-29] Thomson G : Maniola (Epinephile) Jurtina (L.)(Lep. Satyridae) and Its Forms. *Entomol Rec J Variation.* 1969. Reference Source

[ref-30] Uliano-Silva M Nunes JGF Krasheninnikova K : marcelauliano/MitoHiFi: mitohifi_v2.0.2021. 10.5281/zenodo.5205678

[ref-31] UniProt Consortium: UniProt: A Worldwide Hub of Protein Knowledge. *Nucleic Acids Res.* 2019;47(D1):D506–15. 10.1093/nar/gky1049 30395287 PMC6323992

[ref-32] Weir JC and Others : Intraspecific Taxonomy in the Lepidoptera. *Br J Ent Nat Hist.* 2016;29(3):144–54. Reference Source

