# Supplementary material for: The genome sequence of the meadow brown, Maniola jurtina (Linnaeus, 1758)
Source: Wellcome Open Res. 2021 Nov 5;6:296. [Version 1] doi: 10.12688/wellcomeopenres.17304.1 (PMC9971652; doi:10.12688/wellcomeopenres.17304.1)
Supplement: Peer review history

# Peer review: Reviewer response for version 1

**DOI:** 10.21956/wellcomeopenres.19131.r54294

**Published:** 2023-02-15

**Authors:** Zdenek F. Fric

**Affiliations:** 1 Institute of Entomology, Biology Centre CAS, České Budějovice, Czech Republic

This work represents another published genome from the Darwin Tree of Life, now for a satyrinae butterfly *Maniola jurtina*. Thus, now not many other satyrinae butterflies are left and I hope that soon all the remaining species will follow. The rationale is clear, and the methodology is as well (despite not publishing the exact scripts for the pipeline).

 The result is very welcome, *Maniola jurtina* was thoroughly studied throughout Europe regarding its biogeography and a high-quality genome will be a very important step for later genomic studies on a European scale. However, I would welcome here a short discussion comparing these results with already published results by Sing et al. (2020). ^1^ Here, the authors state that they are aware of the study, but looking inside, the results strongly differ. For instance, the genome size reported by Lohse & Wieir states that the genome size is 402 Mb long, whereas Singh et al (2020) reported a genome size exceeding 600 Mb. So please, where is the solution of the puzzle?

Are sufficient details of methods and materials provided to allow replication by others?

Yes

Is the rationale for creating the dataset(s) clearly described?

Yes

Are the datasets clearly presented in a useable and accessible format?

Yes

Are the protocols appropriate and is the work technically sound?

Yes

Reviewer Expertise:

Phylogeny, evolution and ecology of Lepidoptera

I confirm that I have read this submission and believe that I have an appropriate level of expertise to confirm that it is of an acceptable scientific standard.

## References

[1] : De Novo Genome Assembly of the Meadow Brown Butterfly, Maniola jurtina. *G3 (Bethesda)* .2020;10(5) : 10.1534/g3.120.401071 1477-1484 10.1534/g3.120.401071 32161089PMC7202024

# Peer review: Reviewer response for version 1

**DOI:** 10.21956/wellcomeopenres.19131.r54283

**Published:** 2023-01-27

**Authors:** Violaine Llaurens

**Affiliations:** 1 Institut de Systématique, Evolution et Biodiversité, Université des Antilles Muséum National d'Histoire Naturelle, Paris, France; 2 CNRS, Paris, France

Please note that I reviewed this article together with my colleague Dr Manuela Lopez-Villavicencio who has experience in butterfly genome assembly.

 This article reports the genome assembly of a Maniola jurtina female, allowing access to both the autosomes and the W and Z sexual chromosomes. This butterfly observed in Europe in particular, has raised interest for its ecology. The article clearly written and particularly pleasant to read, It shows convincing evidences for a high-quality assembly. The methods for genome assembly, quality test and Hi-C scaffolding are relevant and up-to-date. Maybe the authors could also add the default parameters used during the hifiasm run and particularly if any purging option was used. They may also provide the version of the softwares used, as well as the percentage of BUSCO proteome complete/fragmented.

 The article also describes mitogenome assembly with mito-hifi, therefore providing a complete view about genomic content for this species.

 Given our own interest about butterfly genomes, we would be keen on knowing the genome-wide level of heterozygosity, as well as the TE content per chromosomes and throughout the whole genome.

 Overall, we think the release of this well-assembled and annotated genome is a useful contribution and we recommend the publication of this article.

 Violaine Llaurens & Manuela Lopez-Villavicencio.

Are sufficient details of methods and materials provided to allow replication by others?

Yes

Is the rationale for creating the dataset(s) clearly described?

Yes

Are the datasets clearly presented in a useable and accessible format?

Yes

Are the protocols appropriate and is the work technically sound?

Yes

Reviewer Expertise:

Evolutionary ecology, population genomics.

I confirm that I have read this submission and believe that I have an appropriate level of expertise to confirm that it is of an acceptable scientific standard.

# Peer review: Reviewer response for version 1

**DOI:** 10.21956/wellcomeopenres.19131.r46928

**Published:** 2021-11-17

**Authors:** Julio Rozas

**Affiliations:** 1 Department of Genetics, Microbiology and Statistics & Biodiversity Research Institute (IRBio), University of Barcelona, Barcelona, Spain

Lohse *et al. *reported the chromosome-level assembly of the meadow brown butterfly *Maniola jurtina*. This assembly was obtained using PacBio (76X coverage), 10X genomics, Hi-C data. For the annotation they we also used RNAseq information. We think that this new data are valuable for the scientific community. Here there are some points, nevertheless, that the authors should consider to improve the manuscript.

**Critical Point:**

1. The Sing *et al. *(2020) paper also describes a genome assembly of a *Maniola jurtina* species. Despite that the assembly quality reported in this paper is lower, there are some important aspects that the authors should compare and comment.
  For instance, the great differences between the flow cytometry and the genome assembly for both assemblies (the assembly length in Sing *et al.* is nearly 50% higher than that reported here). How is the length of some close related butterfly species? There is also a great difference in the number of annotated genes between papers.

**Minor Points:**

1. Introduction: The authors comment that there are some four sub-species (perhaps questionable), but they should explain to which supposed subspecies does the sequenced species belongs.
2. Introduction: Moreover, it would be very interesting and informative, especially for non-taxonomists or butterfly systematics, to show a phylogeny that includes (at least) the various Lepidopteran species with the genome sequenced/assembled, and for which the assembly is at the chromosomal-level quality.
3. Table 1: Despite the number of annotated genes are included in the Ensembl database, it should also be interesting to be reported in the ms.

Are sufficient details of methods and materials provided to allow replication by others?

Yes

Is the rationale for creating the dataset(s) clearly described?

Yes

Are the datasets clearly presented in a useable and accessible format?

Yes

Are the protocols appropriate and is the work technically sound?

Yes

Reviewer Expertise:

Comparative genomics

I confirm that I have read this submission and believe that I have an appropriate level of expertise to confirm that it is of an acceptable scientific standard, however I have significant reservations, as outlined above.
